# Apatinib plus etoposide in pretreated patients with advanced triple-negative breast cancer: a phase II trial

**DOI:** 10.1186/s12885-023-10768-8

**Published:** 2023-05-19

**Authors:** Mengru Cao, Hailing Lu, Shi Yan, Hui Pang, Lichun Sun, Chunhong Li, Xuesong Chen, Wei Liu, Jing Hu, Jian Huang, Ying Xing, Ningzhi Zhang, Yingqi Chen, Ting He, Danni Zhao, Yuanyuan Sun, Lin Zhao, Xiaomeng Liu, Li Cai

**Affiliations:** 1 The Fourth Department of Medical Oncology, Harbin Medical University Cancer Hospital, Harbin, China; 2 The First Ward of the Oncology Department, The First Affilliated Hospital of Harbin Medical University, Harbin, China

**Keywords:** Apatinib, Etoposide, Triple negative breast neoplasms, Angiogenesis inhibitors, Clinical trial

## Abstract

**Background:**

Treatment options for pretreated triple-negative breast cancer (TNBC) are limited. This study aimed to evaluate the efficacy and safety of apatinib, an antiangiogenic agent, in combination of etoposide for pretreated patients with advanced TNBC.

**Methods:**

In this single-arm phase II trial, patients with advanced TNBC who failed to at least one line of chemotherapy were enrolled. Eligible patients received oral apatinib 500 mg on day 1 to 21, plus oral etoposide 50 mg on day 1 to 14 of a 3-week cycle until disease progression or intolerable toxicities. Etoposide was administered up to six cycles. The primary endpoint was progression-free survival (PFS).

**Results:**

From September 2018 to September 2021, 40 patients with advanced TNBC were enrolled. All patients received previous chemotherapy in the advanced setting, with the median previous lines of 2 (1–5). At the cut-off date on January 10, 2022, the median follow-up was 26.8 (1.6–52.0) months. The median PFS was 6.0 (95% confidence interval [CI]: 3.8–8.2) months, and the median overall survival was 24.5 (95%CI: 10.2–38.8) months. The objective response rate and disease control rate was 10.0% and 62.5%, respectively. The most common adverse events (AEs) were hypertension (65.0%), nausea (47.5%) and vomiting (42.5%). Four patients developed grade 3 AE, including two with hypertension and two with proteinuria.

**Conclusions:**

Apatinib combined with oral etoposide was feasible in pretreated advanced TNBC, and was easy to administer.

**Clinical trial registration:**

Chictr.org.cn, (registration number: ChiCTR1800018497, registration date: 20/09/2018)

## Introduction

Triple-negative breast cancer (TNBC), characterized by the absence of estrogen receptor (ER), progesterone receptor (PR) and human epidermal growth factor receptor 2 (HER2) expression, accounting for about 15-20% of breast cancers [1]. Compared to other types of breast cancer, TNBC is associated with rapid disease progression, metastasis, and recurrence, which contribute to a poor prognosis [2]. The treatment landscape of TNBC has undergone significant changes with the emergence of new therapeutic options such as PARP inhibitors for patients with BRCA mutations, immunotherapy targeting PD-L1 positive tumors, and antibody-drug conjugates [3]. Despite this, chemotherapy still plays a critical role in the management of TNBC. However, the median overall survival (OS) of chemotherapy was only 2 to 3 years, indicating a greater need for more treatment options [4].

Vascular endothelial growth factor (VEGF)/VEGF receptor (VEGFR) signal pathway plays an important role in tumor angiogenesis, which is essential for tumor growth and spread [5]. Previous studies have demonstrated patients with TNBC showed higher VEGF levels than patients with non-TNBC [6]. As a monoclonal antibody targeting VEGF, bevacizumab alone or in combination with chemotherapy showed promising efficacy for patients with advanced TNBC [7–9]. Apatinib is an oral tyrosine kinase inhibitor (TKI) that inhibits tumor angiogenesis by selectively targeting VEGFR-2 [10]. In 2014, a multicenter phase II trial conducted by Hu et al. [11] found that apatinib alone showed potential benefit for patients with heavily pretreated TNBC, with the median progression-free survival (PFS) of 3.3 months. In another phase II trial, camrelizumab (an immune checkpoint inhibitor) was combined with apatinib in advanced TNBC patients receiving fewer than three lines of systemic therapy [12]. The combination of camrelizumab and apatinib showed favorable efficacy and safety profiles in patients with advanced TNBC, and the median PFS was 3.7 months. Moreover, a phase II trial enrolled patients with locally advanced and metastatic TNBC who failed to at least one line of chemotherapy (including anthracycline or taxane), and administrated camrelizumab plus apatinib and eribulin [13]. The results showed the median PFS was 8.1 months, and the toxicities were manageable.

Etoposide, a cell cycle-specific antitumor drug, inhibits tumor cell proliferation by forming complexes with topoisomerases that interfere with DNA repair [14]. It can be easily administered, and is a viable option for patients with advanced metastatic breast cancer. A previous study has shown that etoposide alone is effective and safe for heavily pretreated patients with metastatic breast cancer [15]. Besides, the combination of etoposide and apatinib also showed potential efficacy for pretreated HER-2 negative metastatic breast cancer, with a median PFS of 6.9 months and a median OS of 20.4 months [16].

Recently, due to the nature of COVID-19 pandemic, many cancer patients are unable to receive intravenous drug therapy on time. Thus, there is an urgent need to explore the accessible and easy-to-use treatment options for advanced TNBC patients. This study aimed to investigate the efficacy and safety of oral apatinib plus etoposide for pretreated patients with advanced TNBC.

## Methods

### Study design and patients

In this phase II trial, pretreated patients with advanced (recurrent or metastatic) TNBC in Harbin Medical University Cancer Hospital were enrolled. ER and PR negative was defined as < 1% in the immunohistochemistry (IHC) nuclear staining. HER-2 negative was defined as IHC staining 0 or 1+, or IHC staining 2 + with fluorescence in situ hybridization or chromogenic in situ hybridization negative. Eligible patients must have received at least one chemotherapy regimen in the advanced setting; had at least one measurable extracranial lesion according to the Response Evaluation Criteria in Advanced Solid Tumors (RECIST) version 1.1; and had an Eastern Cooperative Oncology Group performance status (ECOG PS) score of 0 or 1. Patients with active brain metastases, leptomeningeal disease or uncontrolled hypertension were excluded. Those who had previously taken antiangiogenic agents (except bevacizumab) or etoposide were also excluded.

The study was approved by the Ethics Committee of the Harbin Medical University Cancer Hospital and was performed in accordance with the Declaration of Helsinki. All patients have signed the informed consent form before any procedure. The study was registered at chictr.org.cn (Number: ChiCTR1800018497) on 20/09/2018.

### Procedures

Eligible patients received oral apatinib 500 mg on day 1 to 21, plus oral etoposide 50 mg on day 1 to 14 of a 3-week cycle until disease progression or intolerable toxicities. Etoposide was administered up to six cycles, and patients received apatinib alone after six cycles [17]. In case of grade ≥ 3 hematologic toxicity or grade ≥ 2 non-hematologic toxicity, a dose reduction of apatinib to 250 mg was allowed. Non-hematologic toxicities, including manageable nausea, vomiting, fever with an established cause (e.g., infection or tumor), and grade 3/4 elevated alkaline phosphatase should be treated with symptomatic management without dose suspension or dose reduction. Re-increase of the apatinib dose was not permitted in this study. For the management of treatment toxicity, no more than two dose suspensions per cycle was allowed, and the cumulative duration of dose suspensions should not exceed two weeks per cycle.

Efficacy evaluation was performed using computerized tomography or magnetic resonance imaging every two cycles according to RECIST 1.1 until disease progression or intolerable toxicities. Blood pressure was monitored three times a week. Routine blood, urine, stool, liver and kidney function, electrolytes test and electrocardiogram were performed every cycle. Adverse events (AEs) during the trial were recorded and graded according to the National Cancer Institute Common Terminology Criteria for Adverse Events (NCI CTCAE) version 5.0.

### Endpoints

The primary endpoint was PFS, which defined as the time from patient enrolment to disease progression or death from any cause, whichever came first. The secondary endpoints included OS (defined as the time from patient enrolment to death from any cause), objective response rate (ORR, complete response and partial response), disease control rate (DCR, complete response, partial response and stable disease) and AEs.

### Statistical analysis

Given an accrual period of 12 months, a maximum follow-up time of 18 months, and a two-sided significance level of 0.05, 34 patients with 28 events were allowed detecting an increase of PFS from 2.8 months [18] to 4.3 months with 80% power using a log-rank test. Considering a 20% dropout rate, a total of 40 patients were required. Sample size calculation was determined with PASS 15.0 software (NCSS, LLA, USA).

Descriptive statistical analyses were mainly used. For PFS and OS, patients without document event were censored at last follow-up. The Kaplan-Meier method was utilized to estimate the median PFS and OS, as well as the 95% confidence interval (CI). Subgroup analysis was conducted by liver metastasis, lung metastasis, bone metastasis, prior taxane therapy, prior anthracycline therapy, and prior platinum therapy. Statistical analysis was performed using SPSS version 25.

## Results

### Baseline characteristics of patients

From September 2018 to September 2021, 40 patients with advanced TNBC were included in this study. The median age was 56, with an age range of 27 to 76. Three patients (7.5%) had an ECOG PS score of 0, while 37 (92.5%) had an ECOG PS score of 1. The median number of metastatic sites of all patients was 2 (0–5). Nine (22.5%), nine (22.5%) and thirteen (32.5%) developed lung metastasis, liver metastasis and bone metastasis, respectively. There was no patient had bone-only disease. All patients received previous chemotherapy in the advanced setting, with the median previous lines of 2 (1–5). A total of 24, 32 and 20 patients received prior taxane therapy, anthracycline therapy and platinum therapy, respectively (Table 1).

**Table 1:** Baseline characteristics of patients

Characteristic	Patients (n = 40)
Age, years, median (range)	56 (27–76)
ECOG PS score, n (%)	
0	3 (7.5%)
1	37 (92.5%)
Number of metastatic sites, median (range)	2 (0–5)
Metastatic sites, n (%)	
Lymph nodes	25 (62.5%)
Lung	9 (22.5%)
Liver	9 (22.5%)
Others*	26 (65.0%)
Previous lines of chemotherapy in the advanced setting, median (range)	2 (1–5)
Previous therapy in the advanced setting, n (%)	
Taxane	24 (60.0%)
Anthracycline	32 (80.0%)
Platinum	20 (50.0%)
Others#	34 (85.0%)

* Including 13 patients with bone metastasis, one with brain metastasis, two with skin or soft tissue metastasis, 16 with chest wall metastasis, and two with adrenal metastasis

# Including 15 patients given capecitabine, 13 given cyclophosphamide, ten given gemcitabine, nine given vinorelbine, one given fluorouracil, one given eribulin, one given utidelone and one given raltitrexed

ECOG PS: Eastern Cooperative Oncology Group performance status

### Efficacy endpoints

**Fig. 1:**
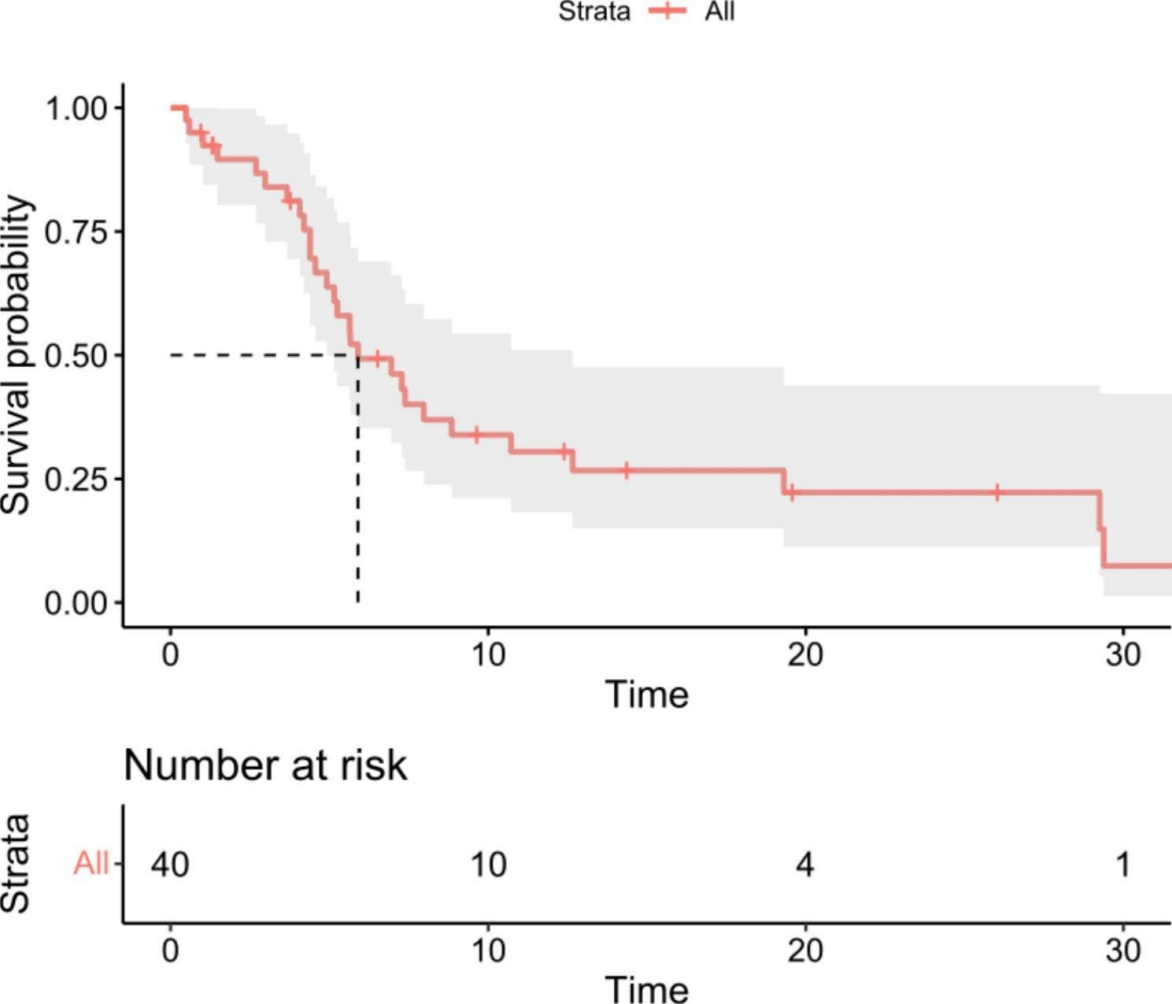
Kaplan-Meier curves of progression-free survival

At the cut-off date on January 10, 2022, the median follow-up was 26.8 (1.6–52.0) months. A total of 30 patients experienced disease progression or death, and 2 patients were still on the treatment. The median PFS was 6.0 (95%CI: 3.8–8.2) months (Fig. 1). Seventeen patients died, and the median OS was 24.5 (95%CI: 10.2–38.8) months. Four patients (10.0%) and 21 patients (52.5%) achieved partial response and stable disease, respectively, with an ORR of 10.0% and a DCR of 62.5%. The subgroup analysis is shown in Table 2. The median PFS was generally consistent among all subgroups. Of the patients included in the study, 42.5% had visceral metastases. In the 22.5% patients who had liver metastases, the median PFS was 7.1 months, in the 22.5% who had lung metastatic disease, the median PFS was 5.3 months, and in the 32.5% who had bone metastatic disease, the median PFS was 7.4 months. In terms of prior treatment with chemotherapeutic agents, 60% of patients had received prior taxane therapy, 80% of patients used anthracyclines, and 50% of patients received platinum. The median PFS for them was 7.1 months, 7.5 months and 8.1 months, respectively.

**Table 2:** Subgroup analysis of progression-free survival (PFS)

Subgroup	Number of patients n (%)	Median PFS, months (95%CI)
All patients	40 (100%)	6.0 (3.8–8.2)
Liver metastasis		
Yes	9 (22.5%)	7.1 (3.8–10.3)
No	31 (77.5%)	5.8 (3.1–8.4)
Lung metastasis		
Yes	9 (22.5%)	5.3 (3.0-7.6)
No	31 (77.5%)	7.1 (5.0-9.2)
Bone metastasis		
Yes	13 (32.5%)	7.4 (3.9–10.9)
No	27 (67.5%)	5.7 (4.2–7.2)
Prior taxane therapy		
Yes	24 (60.0%)	7.1 (5.0-9.1)
No	16 (40.0%)	4.5 (0.8–8.1)
Prior anthracycline therapy		
Yes	32 (80.0%)	7.5 (4.3–10.7)
No	8 (20.0%)	6.0 (3.3–8.7)
Prior platinum therapy		
Yes	20 (50.0%)	8.1 (5.2–11.0)
No	20 (50.0%)	5.0 (3.8–6.2)

CI: confidence interval

### Safety profiles

A total of 38 patients (95.0%) experienced AE of any grade, and most of them were grade 1 or 2. The most common AEs in this study was hypertension (65%), followed by nausea (47.5%), vomiting (42.5%), and hand-foot syndrome (32.5%), which were all well controlled with symptomatic treatment (Table 3). Four cases of grade 3 AE were observed, two of which were hypertension and the other two were proteinuria, all possibly related to the use of apatinib. One patient permanently discontinued apatinib due to grade 3 hypertension, while the other three patients temporarily suspended their treatment with both apatinib and etoposide after experiencing grade 3 AEs. After the AEs were resolved to grade ≤ 1, these patients continued apatinib at a reduced dose of 250 mg while etoposide was resumed at the same dose. Besides, five patients reduced etoposide dose due to AE (mainly nausea, vomiting and hand-foot syndrome). No death related to the treatment was reported.

**Table 3:** Summary of adverse events

Events, n (%)	All patients (n = 40)	
	Any grade	Grade 3
Hypertension	26 (65.0%)	2 (5.0%)
Nausea	19 (47.5%)	0
Vomiting	17 (42.5%)	0
Hand-foot syndrome	13 (32.5%)	0
Abdominal pain	11 (27.5%)	0
White blood cell count decreased	9 (22.5%)	0
Fatigue	7 (17.5%)	0
Skin ulceration	6 (15.0%)	0
Gingival hemorrhaging or pain	6 (15.0%)	0
Proteinuria	4 (10.0%)	2 (5.0%)
Anorexia	4 (10.0%)	0
Alopecia	3 (7.5%)	0
Fever	1 (2.5%)	0

## Discussion

TNBC is associated with poor prognosis, especially for heavily pretreated patients [19]. Novel treatment strategies are urgently needed. In this study, TNBC patients who failed to at least one line of chemotherapy in the advanced setting were enrolled. The results showed that apatinib combined with etoposide was well-tolerated in most patients, and yielded an ORR of 10.0%, and a DCR of 62.5%. The median PFS and OS were 6.0 months and 24.5 months, respectively.

Chemotherapy still plays a critical role in the treatment of TNBC. However, numerous clinical studies and practices show that chemotherapy has limited benefits, with the median OS ranged from 12.5 months to 13.4 months and the median PFS ranged from 2.8 months to 5.3 months for pretreated TNBC [20–23]. Angiogenesis is closely related to tumor growth and metastasis, and antiangiogenic treatment is one of the important strategies in the treatment of metastatic breast cancer [24]. Several studies have demonstrated the promising response rate of the bevacizumab alone or addition to chemotherapy for advanced TNBC [7–9], but there was no significant improvement in OS [25]. These results showed the antiangiogenic agents may benefit TNBC patients.

Apatinib selectively binds to and inhibits VEGFR-2 activity, preventing tumor angiogenesis and inhibiting tumor growth [10]. Several studies have evaluated the role of apatinib in the treatment of advanced TNBC. In a two-stage phase II study, 25 patients with heavily pretreated TNBC received oral apatinib 750 mg daily, and an additional of 59 patients received apatinib 500 mg [11]. The results showed that both doses were effective, and 500 mg is safer than 750 mg. Thus, we chose the 500 mg as the initial dose of apatinib in this study. Besides, recent studies have investigated the role of the combination of camrelizumab and apatinib in pretreated TNBC. The results showed that camrelizumab plus apatinib with and without chemotherapy was effective, with acceptable safety profiles [12, 13]. All these results demonstrated the benefit of apatinib in patients with TNBC.

Antiangiogenic agents combined with chemotherapy may have a synergistic effect, thus improving the prognosis of cancer patients [26]. The combination of apatinib and chemotherapy for patients with cancer has also been evaluated. AEROC trial found apatinib plus oral etoposide is effective and safe for patients with platinum-resistant or platinum-refractory ovarian cancer [17]. Besides, both apatinib and etoposide are oral agents, and thus the treatment can be easily administrated by patients at home without the need for infusion devices [27]. Moreover, a single-arm, phase II trial conducted by Hu et al. [16] evaluated the efficacy and safety of apatinib combined with etoposide in the later-line treatment of HER2-negative metastatic breast cancer. The initial dose of apatinib was 500 mg in patients with ECOG scores of 0–1, and 425 mg in patients with ECOG scores of 2, respectively. According to the study results, ORR was 35.5%, DCR was 87.1%, median PFS was 6.9 months, and median OS was 20.4 months. Compared to our results, the median PFS was prolonged, probably because the hormone receptor-positive patients included in that study had a better prognosis. The study also found that hormone receptor-positive patients (n = 19) had a better PFS (median: 7.4 vs. 3.1 months) than hormone receptor-negative patients (n = 12) [16].

Various other TKIs, either alone or in combination with chemotherapy, have been investigated for treating metastatic TNBC. A randomized clinical trial has compared sorafenib plus capecitabine with capecitabine alone in patients with HER2-negative metastatic breast cancer, and the median PFS was 4.3 months for sorafenib plus capecitabine and 3.0 months for capecitabine in patients with HR-negative disease [28]. A randomized phase II trial compared the efficacy of sunitinib alone and single-agent chemotherapy in patients with pretreated TNBC. The median PFS was 2.0 months in patients receiving sunitinib, and 2.7 months in patients receiving chemotherapy [29]. In addition, a phase III trial also failed to demonstrated the superiority of sunitinib plus docetaxel over docetaxel alone in terms of PFS and OS for the first-line treatment of patients with HER2-negative metastatic breast cancer [30]. The regimens in our study yielded a median PFS of 6.0 months, which was numerically higher than that of these studies. In this study, the median OS was 24.5 months, which was longer than previous studies [20–23]. This may be due to the smaller proportion of patients with visceral metastases, and the possible synergistic effect of apatinib plus etoposide in this study. In order for these findings to be confirmed, further studies are necessary.

Overall, the treatment combination appeared to be well-tolerated in most patients. A total of 65% patients developed hypertension, and most of them were well controlled after anti-hypertension treatment. Nausea and vomiting occurred in 47.5% of patients. Four patients developed grade 3 AEs, including two with hypertension and two with proteinuria. No treatment-related death was reported. Similarly, the most common grade 3 or 4 treatment-related AEs of apatinib plus etoposide were hypertension, fatigue, and thrombocytopenia in previous studies [16]. Our preliminary results suggest that the treatment may be well-tolerated, but further studies with larger sample sizes are needed to confirm these findings.

This study has some limitations. This is a single-arm trial with only 40 patients, so the results may be biased due to the small sample size and lack of a control. Therefore, further phase III randomized controlled trial with increased sample size should be conducted to further investigate the role of apatinib plus etoposide for patients with TNBC.

In conclusion, apatinib in combination with oral etoposide was feasible in pretreated advanced TNBC, with the advantage of ease of administration.

## Data Availability

The original contributions presented in the study are included in the article. Further inquiries can be directed to the corresponding authors.

## List of abbreviations

TNBC: Triple-negative breast cancer
ER: estrogen receptor
PR: Progesterone receptor
HER2: Human epodermal growth factor receptor 2
VEGF: vascular endothelial growth factor
TKI: tyrosine kinase inhibitor
IHC: immunohistochemistry
RECIST: Response Evaluation Criteria in Advanced Solid Tumors
ECOG PS: Eastern Cooperative Oncology Group performance status
AEs: Adverse Events
ORR: Objective response rate
DCR: Disease control rate
